# Size-dependent lymphatic uptake of nanoscale-tailored particles as tumor mass increases

**DOI:** 10.4155/fso.15.60

**Published:** 2015-11-01

**Authors:** Pontus Kjellman, Sarah Fredriksson, Christian Kjellman, Sven-Erik Strand, René in ‘t Zandt

**Affiliations:** 1 Medical Radiation Physics, Department of Clinical Sciences, Lund University, Barngatan 2:1, SE-22185, Lund, Sweden; 2 GeccoDots AB, Lund, Sweden; 3 Hansa Medical AB, Lund, Sweden; 4 Lund University Bioimaging Center, Lund University, Lund, Sweden

**Keywords:** EL4-tumor, MRI, multimodal nanoparticles, sentinel lymph node

## Abstract

**Aim:**

To investigate the size-dependent lymphatic uptake of nanoparticles in mice with rapidly growing syngeneic tumors.

**Materials & methods:**

Mice were inoculated subcutaneously with EL4 lymphoma cells and on day 5 or day 6 of tumor growth, injected peritumorally with either 29 nm or 58 nm of ultra-small superparamagnetic iron oxide nanoparticles. Twenty-four hours later the animals were imaged using MRI.

**Results & conclusion:**

The larger of the two particles can only be detected in the lymph node when injected in animals with 6-day-old tumors while the 29 nm ultra-small superparamagnetic iron oxide nanoparticle is observed on both time points. Tumor mass greatly impacts the size of particles that are transported to the lymph nodes.

## Abstract (lay abstract)

This study aims to improve the way the spreading of certain forms of cancer is detected. The method, known as sentinel lymph node detection, locates and removes the first lymph node that drains the tumor to examine it for cancer cells. The authors want to improve the way this is done by using a new contrast agent based on nanoparticles. Two different sizes of particles were injected around the implanted tumors in mice and imaged with MRI. The results indicate that the mass of the tumor plays a crucial role in the transport of nanoparticles to the lymph node.

**Figure 1.**
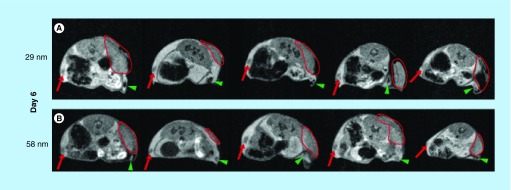
**Axial magnetic resonance-images of animals on day 6 of tumor growth.** **(A)** Animals injected with the 29-nm ultra-small superparamagnetic iron oxide nanoparticles. **(B)** Animals injected with the 58-nm ultra-small superparamagnetic iron oxide nanoparticles. Red arrow indicates lymph node on the contralateral side. Green arrowhead points to sentinel lymph node. Delineated area marks tumor.

**Figure 2.**
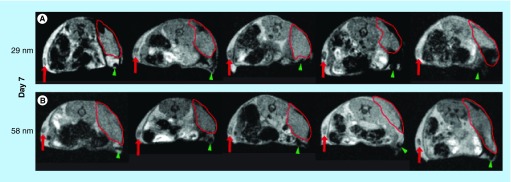
**Axial magnetic resonance-images of animals on day 7 of tumor growth.** **(A)** Animals injected with the 29-nm ultra-small superparamagnetic iron oxide nanoparticles. **(B)** Animals injected with the 58-nm ultra-small superparamagnetic iron oxide nanoparticles. Red arrow indicates lymph node on the contralateral side. Green arrowhead points to sentinel lymph node. Delineated area marks tumor.

**Figure 3.**
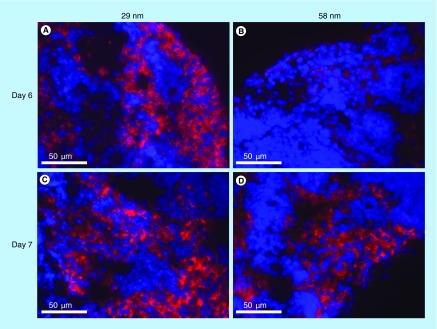
**Fluorescence microscope images of sentinel lymph node 24 h postinjection of ultra small superparamagnetic iron oxide nanoparticles.** The inguinal lymph nodes of all animals were examined. For each lymph node, ten sections (5 μm thick, 15 μm apart) were evaluated (200 in total) for the presence and intensity of ultra-small superparamagnetic iron oxide nanoparticle (USPIO) signal. The areas with the highest USPIO signal were documented and a representation of these is presented here. **(A)** Animal injected with 29-nm USPIOs on day 5 postinoculation. **(B)** Animal injected with 58-nm USPIOs, on day 5 postinoculation. **(C)** 29-nm USPIOs injected on day 6. **(D)** 58-nm USPIOs injected on day 6. Red signal indicates DY-647-labeled USPIOs and blue signal cell nuclei. (Magnification ×40).

**Figure 4.**
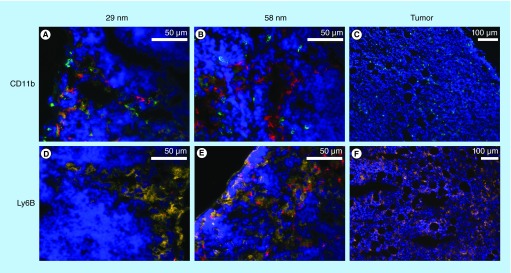
**Immunofluorescence images of sentinel lymph node (A, B, D & E) and tumor (C & F).** Sections in top row **(A–C)** are stained for CD11b (green). Bottom row **(D–F)** is stained for Ly6B (yellow). **(A & D)** represent an animal injected with 29 nm ultra small superparamagnetic iron oxide nanoparticles (USPIOs), **(B & E)** an animal injected with 58 nm USPIOs. Red signal indicates USPIOs, blue signal indicates cell nuclei. (Magnification: sentinel lymph node ×40, tumor ×10).

Over the years many different types of nanoconstructs have been used for *in vivo* imaging, from liposomes and various types of colloidal metal compounds, to quantum dots and solid metal oxide core particles [1]. Different nanostructures are widely used in preclinical investigations, although the biological compatibility and size varies substantially between the various types of construct. Quantum dots, for instance, have shown a high degree of toxicity in cell culture, while the toxic effect in higher biological systems remains a topic of discussion [2–4]. On the other hand, polyethylene glycol (PEG)-coated ultra small superparamagnetic iron oxide nanoparticles (USPIOs) are considered safe also for human diagnostic applications [5]. Another advantage of the PEG-coated USPIOs is that the coating can be easily modified to give the particles a multimodal ability, detectable in various imaging systems [6–8]. The iron oxide core will act as a contrast agent in magnetic resonance imaging, causing a hypo-enhancement in T2/T2*-weighted images. By attaching different functional groups to the coating, such as radionuclides and fluorescent dyes, the particles can be further modified to suit the imaging setup of preference.

At present the clinical practice for sentinel lymph node (SLN) localization is to use a ^99m^Tc-labeled albumin nanocolloid along with a blue dye [9–16]. The SLN is defined as the first lymph node draining the site of a tumor and by investigating the SLN for the presence of tumor cells it is possible to establish if the tumor has started to metastasize. This is a strong prognostic factor and the outcome of the SLN examination governs staging, treatment and prognosis. The contrast agents are administered as a number of injections around the tumor and the SLN is then located and removed, for examination, by surgery. Unfortunately, the small size of the ^99m^Tc-labeled albumin nanocolloid particles allows them to pass beyond the sentinel node and into second-tier nodes, making it difficult to identify the true SLN [15]. However, it has been demonstrated that USPIOs with a size between 10 and 60 nm will accumulate in the SLN, and that the SLN can easily be detected up to 72 h after local USPIO injection [7,17]. Even though the SLN, even at 72 h postinjection, contained a vast amount of particles it could be shown that the amount of particles observed in the SLN is dependent on the size of the particles. An optimal sized nanoparticle would thus increase the sensitivity for SLN detection while keeping the dose of contrast agent low [7,10,12,16,18–20]. Furthermore, it is of importance to determine the optimal nanoparticle size in the diseased state since the size of the lymph node and the lymphatic flow will increase due to the inflammation caused by a rapidly growing tumor [16,21]. It will also be important to determine the optimal size of nanoparticles for use in humans, since the retention of different sizes of particles will vary between species [22]. Hence it might be necessary to use multiple distinct sizes of particles to optimize lymphatic retention in various stages of disease.

In the present investigation two particles, sharing identical solid iron oxide cores but with different thickness of the coating, were injected into mice carrying a syngeneic tumor. Peritumoral injections of USPIOs were used to mimic the scenario of SLN detection in the clinic and the tissue distribution was investigated with MRI on different time points to explore the dynamics of the USPIOs during tumor growth.

## Materials & methods

### USPIOs

The USPIOs were produced as previously described with the exception of the purification of the USPIOs from excess coating material [7]. Briefly; a mixture of iron (III) oxide-hydroxide, octadecene and oleic acid was heated to 323°C for 60 min. The resulting iron oxide cores were coated with poly(maleic anhydride-alt-1-octadecene) and O,O′-bis(2-aminopropyl) polypropylene glycol-block-polyethylene glycol-block-polypropylene glycol through evaporation in a two-phase system. Instead of, as previously, using diafiltration the particles were captured and concentrated on a magnetic separation column (LS-column, Miltenyi Biotec, Bergisch Gladbach, Germany) and washed extensively with 150 mM NaCl. The magnet was removed and the particles were eluted in approximately 1 ml 150 mM NaCl. The size of the particles was determined by dynamic light scattering using a Malvern Zeta Sizer Nano Series (Malvern Instruments Ltd, Worcestershire, UK).

### Tumor model

EL-4 cells (mouse lymphoma) were cultured in RPMI 1640 media, supplemented with 10% new-born calf serum and 1% penicillin/streptomycin, at 37°C, 5% CO_2_. The cells were harvested and resuspended in phosphate buffered saline (PBS) at a cell concentration of 30 × 10^6^ cells/ml. Female C57BL/6 mice (Taconic, Ry, Denmark), weighing approximately 20 g, were anesthetized with isoflurane and injected subcutaneously, in the right flank, with 3 × 10^6^ cells in 0.1 ml of suspension. This tumor model grows very fast [23,24], sometimes resulting in bleeding, necrosis and collapse after as little as 1 week postinoculation. A dramatic increase in the size of the lymph node in the area affected by the tumor is observed at day 6–7 postinoculation [unpublished data]. On day 5 or day 6 postinoculation, one time point before and one during this observed tumor growth, the animals (five per particle) were anesthetized with isoflurane and 0.1 ml USPIOs (340 μg Fe/ml) were administered as four subcutaneous peritumoral injections. Approximately 24 h postinjection of USPIOs, the animals were imaged with MRI (2.4 T, Bruker Avance II, Bruker Biosciences Corporation, MA, USA). The respiratory rate and body temperature was monitored during imaging (S.A. Instruments Inc., NY, USA). After the magnetic resonance (MR) data collection was finished, the animals were sacrificed and the inguinal lymph nodes on the injection side and contralateral side, along with the tumor, were harvested and snap frozen in isopentane. All studies were conducted in accordance with the Swedish guidelines for the use and care of laboratory animals.

### MRI

Optimal image settings were empirically established at an echo time of 6 ms (3D-GE, repetition time: 27 ms, field of view: 60 × 30 × 30 mm^3^, pixel matrix size: 256 × 128 × 128, scan time 16 min 43 s, 4 averages). MR images were evaluated by two scientists independently on the presence of USPIOs in the lymph nodes and tumor of each animal.

### Histology

The lymph nodes were cryo-sectioned in 5 μm and the tumors in 8 μm tissue sections and mounted on Superfrost Plus microscope slides (Thermo Scientific, MA, USA). For evaluation of USPIOs using fluorescence microscopy the tissue sections were fixated in 4% paraformaldehyde and rinsed in PBS. For immunofluorescence staining the slides with frozen tissue sections were immediately fixated with ice-cold acetone and then dried for 30 min, dark at room temperature. The tissue sections were washed and rehydrated in PBS + 0.05% Tween-20, after which the tissue was blocked with 5% rabbit serum in PBS + 0.025% Triton X-100 for 60 min at room temperature. The primary antibody (FITC anti-CD11b [BD Biosciences Pharmigen, CA, USA] and anti-Ly6B.2 [Serotec, Oxford, UK]) was diluted to 2 μg/ml and 20 μg/ml, respectively, in PBS + 0.025% Triton X-100 and incubated with the tissue sections for 60 min at room temperature. Slides were washed in PBS + 0.05% Tween-20 and incubated with the secondary antibody (Alexa Fluor 594 rabbit-anti-rat IgG [Invitrogen, CA, USA]) at 0.4 μg/ml for 60 min, after which the slides were washed in PBS + 0.05% Tween-20.

Cover slips were mounted using ProLong Gold antifade reagent with DAPI (Invitrogen) and the slides were cured over night at room temperature, shielded from light. Fluorescence microscopy was performed using a Zeiss Axiovert 200M microscope (Zeiss, Oberkochen, Germany) equipped with a XBO 75 Xenon lamp and a Hamamatsu ORCA-ER CCD camera (Hamamatsu Photonics K.K., Hamamatsu, Japan). Acquisition and co-registration of fluorescence images was performed using the Volocity software (Improvision, MA, USA).

## Results

### USPIOs

Using the methods previously described [7] two different sized USPIOs were synthesized. A modified purification protocol was implemented; in other words, the particles were purified and concentrated using magnetic separation instead of diafiltration in order to increase the recovery of the particles. Using this new production protocol we established a faster and simpler method, with high reproducibility, for producing the USPIOs. However, the smallest USPIOs generated with this method were significantly bigger than the ones produced with the previous protocol. Despite this, the two USPIO-candidates used in this study were chosen on a stability and difference in size criterion rather than an absolute size criterion. The mean size (±SD), determined by dynamic light scattering, was 29 ± 1.7 nm and 58 ± 4.6 nm for the two USPIOs, respectively.

### MRI

Mice subcutaneously inoculated (day 0) with syngeneic lymphoma cells were on day 5 or day 6 injected peritumorally with 29- or 58-nm-sized USPIOs. Twenty-four hours after USPIO-injection, in other words, on day 6 or day 7, the tissue distribution of particles was investigated using MRI. The mean size of the tumors was 0.13 ± 0.10 cm^3^ on day 6 and 0.44 ± 0.13 cm^3^ on day 7, a significant size increase between the two days. When injected on day 5, USPIOs could be detected in the SLN of four out of five animals given the 29-nm particles (Figure 1A) but only one out of five animals injected with the 58 nm particles (Figure 1B). However, when injected on day 6 post-tumor inoculation, USPIOs were detectable in the SLN of all animals, regardless of the size of the injected particles (Figure 2). Particles were not detectable by MRI in the contralateral lymph nodes of any of the tested animals. Furthermore, 24 h after peritumoral injection a substantial amount of USPIOs were still detectable at the injection site in all tested animals. However, using MRI, there was no detectable influx of USPIOs into the actual growing tumor mass.

### Histology

The targeting of particles to the SLN was further evaluated using fluorescent microscopy. The distribution observed by microscopy examination agreed well to the data obtained with MRI. The USPIOs were predominantly located in the subcapsular sinus of the lymph nodes, with minor amount observed in the deeper situated areas. Due to the lower detection limit in fluorescent microscopy compared with MRI, USPIOs could be detected in the sentinel lymph nodes of all of the animals, although at varying amounts. High amounts of USPIOs could be detected on both day 6 and day 7 post-tumor inoculation in the animals injected with the 29 nm particles (Figure 3A & C). However, on day 6 in animals injected with the 58 nm particles, only minute amounts of USPIOs could be detected in the SLN (Figure 3B) but on day 7 the fluorescence signal is on par with that seen in the animals injected with the 29 nm particles (Figure 3D). Interestingly, on day 7 and regardless of particle size there are several individuals where low levels of particles can be detected in the lymph node on the contralateral side. Furthermore, particles can also be detected inside cells in the periphery of some of the tumors, especially at day 7 of tumor growth.

The immunohistochemical staining of the lymph nodes revealed USPIOs inside both CD11b-positive and Ly6B-positive cells. The signal from the 29-nm particles is closely associated to the CD11b-positive cells (Figure 4A). Particles could also be seen in Ly6B-positive cells, although the signal observed from the CD11b-positive cells is more pronounced (Figure 4D). For the 58-nm particles, however, the particle signal is not as closely associated to the CD11b-positive cells as for the 29-nm particles (Figure 4B). Instead the USPIOs could be detected in numerous CD11b-negative cells and there is a strong fluorescence signal from particles in Ly6B-positive cells (Figure 4E). The tumor sections show both CD11b- and Ly6B-positive cells scattered throughout the tumor (Figure 4C & F). The cells, at the periphery of the tumor, that contained USPIOs were from both CD11b-positive and Ly6B-positive cell lineages.

## Discussion

The model for studying the retention of USPIOs in the SLN used in this paper, mimics the method in which contrast agents for sentinel lymph node diagnosis are administered in the clinic, in other words, the USPIOs are injected around the tumor and then allowed to move to the SLN before imaging. However, contrary to the contrast agents used in the clinic, the USPIOs used in our study have a retention time in the SLN exceeding 24 h, allowing administration and imaging of the contrast agent at least 24 h prior to surgery. Two major processes influence the retention of USPIOs in the lymph nodes, transport of USPIOs by the lymph from the injection site and uptake by cells, either at the injection site with subsequent migration to the lymph nodes or by resident cells in the lymph node.

Two factors affect the transport of USPIOs in our study; the subcutaneous pressure on the fluid after injection and the inflammatory response to the tumor. It has been shown that lymphatic clearance of subcutaneously administered particles to the flank of rodents is poor [25]. A major contributing factor of this is the volume of the subcutaneous space. An increase in subcutaneous pressure will widen the channels leading to the lymphatic vessels and will force elevated levels of fluid into the lymphatic system. A comparison can be made to an injection that is administered in the paw of the animal, which is a method for studying lymphatic uptake in healthy animals. The subcutaneous space is much more limited in the paw than in the flank and a smaller injection volume will be sufficient to elevate the subcutaneous pressure and hence the lymphatic uptake. In a subcutaneously growing tumor in the flank of the mouse, the subcutaneous space is larger compared with the paw and the pressure-increase that the injection causes will only have a marginal effect on lymphatic uptake. However, in this EL4-lymphoma tumor model, the connective tissue surrounding the tumor is compressed when the solid tumor grows. As can be seen in the MR-images the USPIOs are present in this connective tissue, even 24 h after the injection. It can be anticipated that the increasing pressure on the connective tissue by the growing tumor enhances the pressure exerted by the particle injection and hence the lymphatic uptake.

Another factor contributing to the transport of USPIOs through the lymphatic system is the increase in the lymphatic flow from the site as well as the recruitment of leukocytes due to the inflammation caused by the growing tumor [16,21]. This increase in lymph flow enables the larger particle to move into the lymphatics and to the SLN.

The uptake and retention of USPIOs in lymph nodes is also affected by the uptake of USPIOs by cells, studied using immunohistochemistry. Ly6B antibodies stain immature and mature neutrophils and monocytes but do not stain macrophages, lymphocytes, eosinophils, mast cells or erythroid cells, while CD11b is found on macrophages, Kupffer cells as well as granulocytes and dendritic cells [26]. EL-4 cells are generally considered as being both CD11b- and Ly6B-negative [27]. The phagocytosis of the 58-nm particles differs from that of the 29-nm particles in the type of cells where the particles can be detected. For the 29-nm particle a clear correlation between USPIO fluorescence and CD11b-positive cells (most likely macrophages) can be seen. The 58 nm particles are also to some extent found in these CD11b-positive cells but the signal is not as clearly correlated with the CD11b-staining and the 58-nm USPIOs are found also in other cell types. When the tissue sections are stained for Ly6B a clear co-localized signal can be observed between these Ly6B-positive cells and the 58-nm USPIOs. The 29-nm particles do not display the same degree of co-localization. The USPIOs were predominantly located in the subcapsular sinus of the lymph nodes, with minor amount observed in the deeper situated areas [17,28].

Others have shown that PEG increases the ability of particles to evade phagocytosis by macrophages [29,30]. The 58-nm particles have a thicker PEG-layer than the 29-nm particles. This could explain the differences seen between the two USPIOs in uptake by CD11b-postive cells. The outer-most layer of the 58-nm particles consists of a methylated PEG-layer, while the 29-nm particles have a surface layer with residual amino-PEG groups. As lymphatic fluid has a slightly alkaline pH these groups should be uncharged when the particles enter the lymphatics [31]. However, this potentially minor difference in surface chemistry is unlikely to influence the fate of the USPIOs in the lymphatics [32].

The increased perfusion of lymph fluid, in and around the tumor, might explain why USPIOs are detected in the contralateral lymph node of some of the animals imaged on day 7, of tumor growth. As the injections are placed in four peritumoral locations, the larger size of the tumor will force the injection to be placed closer to the back midline of the mice. The increased flow of lymph as well as the close proximity to the back midline might cause fluid, particles and cells to ‘spill over’ to the contralateral side, leading to the findings of USPIOs in this ‘control node’. The finding of USPIO-containing cells in the periphery of the tumors might also be an effect of the ‘age’ of the tumor. The rapidly growing tumor causes a recruitment of leukocytes, which have the capacity to phagocytize particles in the subcutaneous space [26,27,33]. This recruitment was illustrated by numerous of both CD11b- and Ly6B-positive cells with ingested USPIOs identified by histology.

Even though it is difficult to quantify the amount of USPIOs using MRI, it is clear that, on day 6 of tumor growth, there is a difference in the amount of particles in the SLN between the two particle sizes. The same difference was observed using fluorescence microscopy. When the tumors were allowed to grow for an additional day the difference in USPIO uptake, between the two sizes, can no longer be detected and both are readily observed in the SLN.

## Conclusion

In conclusion this study has shown that the lymphatic uptake of USPIOs injected peritumorally increases as the burden on the lymphatics is increased by the growing tumor and that the size of the particle has a significant influence on the uptake in the SLN of tumor bearing animals. Future work will focus on the dynamics and multimodal nature of the two different particles in the same animal as well as studying the dynamics of uptake of nanostructures in tumor models with growing metastases in the SLN. Finally, the size-dependent pharmacokinetics, indicated by this study, should be explored as a possible criterion for cancer staging.

## Future perspective

Multimodal nanoparticle-based contrast agents, like the ones used in the article, are showing great promise to advance the field of sentinel lymph node detection. Taking the results from this investigation into account it is possible that using a nanoparticle of a certain size, or even mixture of particles of different carefully selected sizes, can be used not only for detection of the sentinel lymph node but also staging the cancer. If a particle, with a size larger than a specific threshold, will only be transported to the sentinel lymph node after the tumor reaches a certain stage, the detection of these particles in the SLN would indicate the staging of the tumor. By labeling the nanoparticles with different markers it would be possible to distinguish the different sizes from the mixture in the patient. This would reduce the time needed for each procedure, as the USPIOs can be administered and imaged days ahead of surgery, allowing for more procedures being performed and thereby reducing costs. With a nanoparticle that also allows staging of the tumor, numerous patients could also be spared unnecessary lymph node surgery. It is quite possible that the treatment of several other types of cancer could benefit from this type of procedure and contrast agent, not only breast cancer and malignant melanoma, which are the most frequently used at present.

**Executive summary**

There is a need to develop a new contrast agent for sentinel lymph node diagnosis, that only labels the sentinel lymph node (SLN), remains in the lymph node in excess of 24 h and can be visualized by multiple medical imaging modalities. Nanoparticles show great promise to constitute this new contrast agent.
Mice inoculated with rapidly growing EL4 tumors were injected peritumorally with either of two sizes of ultra small superparamagnetic iron oxide nanoparticles (USPIOs). The particles were administered on either day 5 or day 6 of tumor growth.
The retention of USPIOs in the SLN was visualized *in vivo* with MRI and *ex vivo* with fluorescence microscopy.
The uptake and retention of USPIOs in the SLN of tumor bearing mice is shown to be size-dependent, both on account of the size of the tumor and the nanoparticles.
A smaller particle is more readily taken up into the lymphatics, when injected peritumorally. However, as the tumor grows the transport of larger particles to the SLN is increased.
It is shown that even a rather small difference in particle size, approximately 30 nm, has a significant effect on the lymphatic uptake.
